# Supplementary material for: MetaRibo-Seq measures translation in microbiomes
Source: Nat Commun. 2020 Jun 29;11:3268. doi: 10.1038/s41467-020-17081-z (PMC7324362; doi:10.1038/s41467-020-17081-z)
Supplement: Supplementary file 10 — Supplementary Data 7 [file 41467_2020_17081_MOESM10_ESM.zip › File2/Confidence_High_Taxonomy/67063_out.krona.html]

Javascript must be enabled to view this page.

members
magnitude
magnitudeUnassigned
count
unassigned
taxon
rank

67063\_out

5


SRS053356\_contig\_number\_10235
1

3
superkingdom
2

phylum
1
201174

84992
class
1

84993
1
order

84994
1
family

species
1

SRS098571\_contig\_number\_54469
2024894

200795
2
phylum


SRS051031\_contig\_number\_contig-100\_1579.184401SRS1041145\_contig\_number\_16127
1797616
species
2

2759
1
superkingdom

1
kingdom
4751

subkingdom
1
451864

1
phylum
4890

subphylum
1
147538

1
class
147545

subclass
1
451871

33183
1
order

1
genus
38946

SRS023914\_contig\_number\_20933
